# A Comprehensive Review of Extra Corporeal Membrane Oxygenation: The Lifeline in Critical Moments

**DOI:** 10.7759/cureus.53275

**Published:** 2024-01-31

**Authors:** Sindhu Geetha, Neeta Verma, Vivek Chakole

**Affiliations:** 1 Anaesthesiology, Jawaharlal Nehru Medical College, Datta Meghe Institute of Higher Education and Research, Wardha, IND

**Keywords:** remote monitoring, miniaturization, cardiac failure, respiratory failure, critical care, extracorporeal membrane oxygenation (ecmo)

## Abstract

Extracorporeal membrane oxygenation (ECMO) has evolved into a pivotal intervention in critical care, offering a lifeline for patients facing severe respiratory or cardiac failure. This review provides a comprehensive exploration of ECMO, spanning its definition and historical background to its contemporary advancements and ongoing impact in critical care. The versatility of ECMO in addressing diverse critical conditions, careful patient selection criteria, and the nuanced management of complications are discussed. Advances in technology, including miniaturization, novel circuit designs, and the integration of remote monitoring, showcase the evolving landscape of ECMO. The review underscores the ongoing impact of ECMO in improving survival rates, enhancing mobility, and enabling remote expertise. As a symbol of hope and innovation, ECMO's lifesaving potential is evident in its ability to navigate the complexities of critical care and redefine the boundaries of life support interventions.

## Introduction and background

Extracorporeal membrane oxygenation (ECMO) has emerged as a pivotal life-supporting intervention in critical care, providing a lifeline for patients facing severe respiratory or cardiac failure. This review delves into the intricate facets of ECMO, beginning with a foundational exploration of its definition, historical evolution, and crucial significance in critical care [1]. ECMO is an advanced life-support technique that involves temporarily taking over the functions of the heart and lungs through an external circuit. This extracorporeal system consists of a pump that propels blood through an artificial lung (oxygenator), allowing for the removal of carbon dioxide and the infusion of oxygen before returning the blood to the patient's circulatory system [2].

The overarching goal of ECMO is to provide temporary support to failing organs, thereby allowing the patient's native organs to rest and recover. This intervention is particularly vital in situations where conventional therapies fall short, such as in cases of severe acute respiratory distress syndrome (ARDS) or refractory cardiac failure [3]. The roots of ECMO trace back to the 1950s, marked by pioneering efforts in extracorporeal circulation and oxygenation. However, it was in the 1970s that ECMO gained notable attention with successful applications in neonates. The landmark "Toronto-Belleville ECMO" trial in 1979 demonstrated improved survival rates in infants with respiratory failure, sparking a surge of interest in the technique [4].

Since then, ECMO has undergone significant refinement, expanding its applications beyond neonatal care to include pediatric and adult populations. The historical trajectory underscores the iterative advancements that have shaped ECMO into a versatile and indispensable tool in critical care [5]. The importance of ECMO in critical care cannot be overstated. In scenarios where traditional therapies are insufficient, ECMO steps in as a bridge, providing a window for recovery or facilitating the transition to more definitive treatments such as organ transplantation. It is a dynamic intervention capable of sustaining life while allowing time to resolve underlying pathologies [6]. Moreover, ECMO's role extends beyond respiratory and cardiac support; it has proven valuable in diverse clinical scenarios, including trauma, sepsis, and post-cardiotomy shock. As technology and expertise in ECMO continue to advance, its relevance in critical care settings is poised to expand, offering new avenues for managing complex medical conditions [6].

## Review

Types of ECMO

Veno-Venous ECMO

Veno-Venous ECMO (VV-ECMO) is a configuration primarily designed to provide respiratory support. In this modality, blood is drained from a vein, typically the inferior or superior vena cava, and is then oxygenated outside the body before being returned to the venous system. By circumventing the lungs, VV-ECMO allows for enhanced oxygenation and carbon dioxide removal, making it particularly effective in cases of severe respiratory failure, such as ARDS [7]. This technique offers a more focused intervention on the respiratory aspect of critical care, providing a means to alleviate strain on compromised lungs and facilitate healing. The ability to adjust blood flow rates and oxygenation levels allows for a personalized approach to patient management [8].

Veno-Arterial ECMO

Veno-Arterial ECMO (VA-ECMO), on the other hand, encompasses both respiratory and circulatory support. It involves withdrawing blood from a vein, oxygenating it, and returning it to an artery, effectively bypassing the heart and lungs. VA-ECMO is instrumental in profound cardiac failure or cardiogenic shock, providing oxygenation and mechanical circulatory support [9]. This modality serves as a bridge to recovery for patients with severe cardiac conditions, allowing the heart to rest and heal while ensuring adequate oxygenation of vital organs. The dual capability of addressing respiratory and cardiac functions makes VA-ECMO a versatile option for critically ill patients with complex pathologies [10].

Extracorporeal Life Support

Extracorporeal life support (ECLS) is a broader term encompassing various extracorporeal support systems, including VV-ECMO and VA-ECMO. ECLS represents a comprehensive approach to life support, acknowledging the dynamic interplay between respiratory and circulatory functions [11]. ECLS systems are adaptable and can be configured based on the patient's needs, making them suitable for various critical conditions. The term ECLS is often used interchangeably with ECMO, reflecting the overarching role of these systems in providing a lifeline for patients facing life-threatening medical crises [12].

Indications for ECMO

Respiratory Failure

One of the primary indications for ECMO is severe respiratory failure, often refractory to conventional mechanical ventilation. Conditions such as ARDS, severe pneumonia, or other forms of lung injury can lead to profound hypoxemia and hypercapnia that may be challenging to manage with standard ventilatory strategies [13]. ECMO provides a crucial avenue for oxygenation and carbon dioxide removal, allowing the lungs to rest and recover. VV-ECMO is particularly well-suited for cases with respiratory issues. By temporarily assuming the oxygenation function of the lungs, ECMO serves as a bridge to recovery for patients struggling with severe respiratory compromise [9].

Cardiac Failure

In situations of cardiac failure or cardiogenic shock where the heart's pumping capacity is severely compromised, VA-ECMO becomes instrumental. VA-ECMO provides oxygenation and circulatory support by bypassing the heart and pumping oxygenated blood directly into the arterial system [14]. This form of ECMO serves as a bridge to recovery for patients awaiting cardiac interventions or as a temporary support measure while the heart heals. It plays a pivotal role in stabilizing patients in critical conditions, allowing time for the underlying cardiac pathology to be addressed and managed [15].

Bridge to Transplant

ECMO is often employed as a bridge to transplantation in cases where organ transplantation, either pulmonary or cardiac, is deemed necessary. It serves as a life-sustaining intervention while patients await suitable donor organs. By providing temporary support, ECMO enhances the likelihood of successful transplantation by maintaining vital organ function until a compatible organ becomes available [16]. The bridging function of ECMO is not limited to transplantation alone; it can also be employed as a bridge to recovery, enabling patients to regain organ function and stability before more definitive interventions are needed [15].

Other Critical Conditions

Trauma: In cases of severe trauma where traditional circulatory and respiratory support methods may prove insufficient, ECMO emerges as a valuable intervention. Trauma can lead to profound hemodynamic instability, compromising respiratory and circulatory functions. In this context, ECMO serves as a temporary life support system, providing a dual mechanism of respiratory oxygenation and circulatory assistance. By taking over the functions of the heart and lungs, ECMO allows vital organs to rest and recover, offering a crucial bridge until the patient stabilizes or more definitive interventions can be pursued [17].

Sepsis: ECMO plays a role in managing cases of refractory septic shock where traditional therapies fail to restore hemodynamic stability. In sepsis, an overwhelming immune response to infection can lead to cardiovascular collapse and respiratory failure. ECMO provides comprehensive support by simultaneously addressing both respiratory and circulatory compromises. The extracorporeal circuit facilitates efficient gas exchange, aiding in oxygenation, while the pump assists the heart in maintaining circulatory perfusion. By offering a reprieve to the overwhelmed native systems, ECMO stabilizes the patient and creates a window for resolving the underlying septic process [18].

Post-cardiotomy shock: Post-cardiotomy shock is a critical complication that can arise after cardiac surgery, characterized by profound circulatory instability. ECMO is a vital support mechanism when conventional methods fail to restore adequate cardiac output and tissue perfusion. The surgery itself may place significant stress on the heart, leading to an inability to maintain circulatory function. VA-ECMO, in particular, can provide respiratory and circulatory support, allowing the heart to rest and recover. By temporarily offloading the heart's workload, ECMO becomes a crucial tool in stabilizing patients in the immediate postoperative period, enhancing the chances of a successful recovery and reducing the risk of further complications [19].

ECMO components and circuit

Oxygenator

The oxygenator is the heart of the ECMO circuit and facilitates gas exchange outside the body. This vital component removes carbon dioxide and oxygen infusion into the patient's blood. Modern oxygenators are typically made of biocompatible materials and utilize a network of hollow fibers to create an interface between the patient's blood and the oxygen-rich sweep gas [20]. Oxygenators come in various designs, including membrane and bubble types, offering distinct advantages in clinical scenarios. The efficiency and biocompatibility of the oxygenator are critical factors influencing the overall success of ECMO therapy [20].

Pump

The pump is an essential mechanical component that propels blood through the ECMO circuit. It ensures a continuous and controlled flow, allowing for the efficient exchange of gases in the oxygenator. Peristaltic and centrifugal pumps are commonly used in ECMO systems, each with unique characteristics [20]. The pump's ability to maintain adequate blood flow is paramount, as it directly influences the ECMO's oxygenation and circulatory support. The pump's speed can be adjusted to meet the patient's physiological needs, providing a dynamic and personalized approach to life support [20].

Cannulation

Cannulation involves the insertion of tubes (cannulas) into major blood vessels, establishing the connection between the patient and the ECMO circuit. The choice of cannulation sites depends on the specific clinical scenario and the type of ECMO employed [10]. For VV-ECMO, cannulas are typically placed in the large veins, such as the superior or inferior vena cava. In venous-arterial ECMO (VA-ECMO), cannulation involves both venous and arterial sites, allowing circulatory support. Cannulation is a delicate procedure requiring precision to optimize blood flow and minimize complications [10].

Circuit Monitoring

Continuous monitoring of the ECMO circuit ensures its optimal function and patient safety. Monitoring encompasses a range of parameters, including blood flow rates, gas exchange efficiency, and pressure measurements within the circuit. Regularly assessing these parameters allows healthcare professionals to identify deviations or complications promptly [21]. In addition to circuit-specific monitoring, comprehensive patient monitoring is crucial. This involves continuous assessment of vital signs, laboratory values, and imaging studies to gauge the overall response to ECMO therapy. Regular multidisciplinary communication and collaboration are essential to promptly address any emerging issues [21].

Patient selection and management

Criteria for ECMO Initiation

Severe respiratory or cardiac failure: When conventional therapeutic approaches prove insufficient for maintaining adequate respiratory or cardiac function, ECMO becomes a critical intervention. In cases of severe respiratory failure, where oxygenation and carbon dioxide removal are compromised, or in cardiac failure with a compromised ability to pump blood effectively, ECMO temporarily assumes the vital functions of the heart and lungs. By providing extracorporeal support, ECMO allows the patient's native organs to rest and recover, offering a bridge to potential improvement or more definitive interventions [22].

Failure of conventional mechanical ventilation: Conventional mechanical ventilation may encounter limitations in cases of refractory hypoxemia or hypercapnia despite optimal settings. In such situations, ECMO is an advanced respiratory support strategy, offering a more robust means of oxygenation and carbon dioxide removal. By bypassing the lungs, ECMO can augment or even replace the function of mechanical ventilation, providing a vital alternative for patients who fail to respond to traditional respiratory support measures [23].

Bridge to transplant or recovery: ECMO plays a pivotal role as a bridge to either organ transplantation or patient recovery. In scenarios where patients are awaiting organ transplantation, ECMO serves as a life-sustaining measure, maintaining vital organ function until a suitable donor organ becomes available. Additionally, for patients experiencing severe but potentially reversible organ failure, ECMO offers a bridge to recovery by providing temporary support. This allows time for resolving the underlying pathology or the patient's condition to improve sufficiently to sustain life without ECMO [24].

Post-cardiotomy shock: Post-cardiotomy shock, a critical condition following cardiac surgery, can lead to circulatory collapse or an inability to wean from cardiopulmonary bypass. ECMO becomes an essential tool in stabilizing patients during this postoperative period. By providing circulatory support, ECMO assists the heart in maintaining perfusion and allows for a gradual weaning process, giving the heart the necessary time to recover from the stress of surgery [25].

Trauma or sepsis with circulatory collapse: In situations of trauma or sepsis where there is a rapid onset of circulatory collapse, ECMO becomes a life-saving intervention. Trauma may lead to severe hemorrhage and cardiovascular instability, while sepsis can cause refractory shock. ECMO provides respiratory and circulatory support, stabilizing the patient in critical conditions where traditional methods may not be sufficient. By temporarily taking over vital functions, ECMO creates a therapeutic window for addressing the underlying trauma or septic process [17].

Pre-ECMO Assessment

Imaging studies: Chest X-rays and computed tomography (CT) scans: Imaging studies are crucial in the pre-ECMO assessment. Chest X-rays provide a detailed view of lung conditions, helping clinicians evaluate the extent of damage, the presence of infiltrates, and the overall lung parenchymal status. CT scans offer a more comprehensive assessment, aiding in identifying specific lung pathologies and guiding decisions related to cannulation. The information gleaned from these imaging modalities assists in determining the most suitable approach for ECMO initiation and optimizing support for respiratory failure [26].

Echocardiography: Echocardiography is a fundamental component of the pre-ECMO assessment, particularly in cases where VA-ECMO is considered. This imaging modality allows for real-time visualization of cardiac function, helping clinicians assess the heart's pumping capacity, identify structural abnormalities, and evaluate the overall cardiac performance. The findings from echocardiography guide decisions about the need for circulatory support and inform cannulation strategies. In VA-ECMO, precise placement of venous and arterial cannulas is crucial, and echocardiography provides valuable insights to optimize the configuration and efficacy of ECMO support [27].

Laboratory values: Comprehensive blood tests are essential in the pre-ECMO assessment, offering valuable insights into the patient's physiological status. Coagulation profiles, including measures such as activated partial thromboplastin time (aPTT) and international normalized ratio (INR), inform decisions about anticoagulation strategies during ECMO therapy. Renal function tests, such as serum creatinine levels, guide the assessment of kidney function, which is particularly important given the potential impact of ECMO on renal perfusion. Overall, metabolic profiles help understand the broader physiological context, influencing decisions related to anticoagulation and blood management strategies tailored to the individual patient [28].

Infection screen: Identifying and addressing underlying infections is critical to the pre-ECMO assessment. Infections can not only contribute to the need for ECMO but may also pose complications during therapy. Rigorous screening for infections, including blood cultures and other relevant tests, allows for the early detection and targeted treatment of infectious processes. This proactive approach is essential in preventing complications and optimizing the overall success of ECMO therapy [29].

Neurological assessment: Neurological assessment is integral to evaluating the patient's baseline neurological status and predicting the likelihood of complications during ECMO. This assessment evaluates mental status, pupillary reactions, and motor responses. Neurological status informs decisions about the appropriateness of ECMO, especially considering the potential risks of neurological complications associated with the therapy. A thorough understanding of the patient's neurological condition aids in tailoring ECMO management strategies to minimize the risk of adverse neurological outcomes [30].

ECMO Cannulation Process

VV-ECMO cannulation: VV-ECMO is characterized by the placement of cannulas in large veins, typically the superior or inferior vena cava. This cannulation approach provides respiratory support by extracting deoxygenated blood from the venous system, oxygenating it externally through the ECMO circuit, and then returning the oxygenated blood to the venous system. By bypassing the lungs this way, VV-ECMO supports gas exchange and oxygenation, allowing the patient's lungs to rest and recover from severe respiratory failure [31].

VA-ECMO cannulation: VA-ECMO involves a dual cannulation strategy, incorporating both venous and arterial access points. The venous drainage cannula is typically placed in the right atrium, allowing the extraction of deoxygenated blood. This blood is pumped through the ECMO circuit, which undergoes oxygenation. The oxygenated blood is returned to the patient's arterial system via the arterial return cannula, often inserted into the aorta. VA-ECMO provides respiratory and circulatory support, bypassing the heart and lungs to oxygenate blood and assist with circulatory perfusion. This comprehensive support is particularly beneficial in cases of combined respiratory and cardiac failure, offering a versatile intervention for critically ill patients. The choice of cannulation strategy is guided by the patient's specific clinical needs and the ECMO therapy goals [10].

Anticoagulation and Blood Management

Anticoagulation protocols: Tailored anticoagulation protocols are essential for ECMO therapy, customized based on the patient's coagulation profile. These protocols prevent clot formation within the ECMO circuit, ensuring optimal function. Regularly monitoring coagulation parameters, such as aPTT and international normalized ratio (INR), is conducted to maintain therapeutic anticoagulation levels. The delicate balance between preventing clotting and avoiding bleeding complications requires vigilant adjustment of anticoagulation levels throughout the ECMO support period [32].

Heparin or alternative agents: Heparin, a traditional anticoagulant, is commonly employed in ECMO therapy. However, in cases where patients develop heparin-induced thrombocytopenia or exhibit other contraindications to heparin use, alternative anticoagulant agents may be considered. These alternatives, such as direct thrombin or anti-factor Xa inhibitors, provide options for maintaining anticoagulation while mitigating the risks associated with heparin in specific patient populations [33].

Blood product management: Closely monitoring hemoglobin levels and platelet counts during ECMO therapy is integral to blood product management. The mechanical forces within the ECMO circuit can contribute to hemolysis, leading to decreased hemoglobin levels. Platelet counts may also be affected due to the interaction with the extracorporeal circuit. Regular assessments guide transfusion decisions, ensuring patients receive appropriate blood products to address anemia or thrombocytopenia. Additionally, ECMO-associated hemolysis may necessitate additional interventions, emphasizing the importance of continuous monitoring and proactive management [34].

Thromboembolic risk: The risk of thromboembolic events is elevated in patients undergoing ECMO support due to the extracorporeal nature of the circuit. Prophylaxis against thromboembolic events is crucial to prevent complications such as circuit clotting, emboli, or ischemic events. Anticoagulation and meticulous monitoring of the ECMO circuit and the patient's overall clinical status are implemented to mitigate thromboembolic risk. Strategies for thromboprophylaxis may involve adjusting anticoagulation levels, optimizing blood flow rates, and employing antithrombotic agents when clinically indicated [35].

Complications and challenges

Bleeding and Thrombosis

Bleeding: Anticoagulation is necessary for ECMO to prevent clotting within the circuit, but it predisposes patients to bleeding complications. Balancing the need for anticoagulation with the risk of bleeding is a delicate challenge. Monitoring for signs of bleeding, including at cannulation sites or in other organs, is crucial. Adjustments to anticoagulation levels and protocols may be necessary based on individual patient responses [33].

Thrombosis: Despite anticoagulation, thrombotic complications can occur within the ECMO circuit, potentially leading to device malfunction or embolic events. Regular monitoring of circuit integrity, along with imaging studies, aids in the early detection of thrombosis. Strategies to mitigate thrombotic risk include maintaining optimal blood flow rates, meticulous anticoagulation management, and employing circuit changes when necessary [36].

Infection

Cannula-related infections: The insertion and presence of cannulas provide potential entry points for infections. Rigorous aseptic techniques during cannulation and ongoing monitoring for signs of infection, such as fever or elevated inflammatory markers, are essential. Timely administration of appropriate antibiotics is crucial in managing ECMO-related infections [37].

Systemic infections: ECMO patients, particularly those with prolonged support, are susceptible to systemic infections due to foreign materials in the circuit. Close surveillance for signs of sepsis and proactive infection control measures are imperative in minimizing the risk of complications [38].

Hemolysis

The mechanical forces exerted on blood within the ECMO circuit can lead to hemolysis, the breakdown of red blood cells. Hemolysis can result in anemia, jaundice, and potential complications such as renal dysfunction. Monitoring for signs of hemolysis, including elevated lactate dehydrogenase (LDH) levels and hemoglobinuria, guides interventions such as adjustments to pump speed or circuit changes to mitigate the risk of ongoing hemolysis [39].

Neurological Complications

Cerebral emboli: The risk of cerebral emboli is a critical consideration in ECMO therapy. The extracorporeal circuit introduces the potential for embolic events, which can adversely affect cerebral perfusion and lead to neurological complications. To mitigate this risk, a multifaceted approach is essential. Monitoring neurological status involves regular assessments of mental status, pupillary reactions, and motor responses. Neuroimaging studies, such as CT scans or magnetic resonance imaging (MRI), may be employed to detect and assess the extent of cerebral emboli. Additionally, optimizing anticoagulation levels is crucial to preventing clot formation within the ECMO circuit, reducing the likelihood of embolic events, and preserving cerebral perfusion [40].

Hypoperfusion and ischemia: Inadequate perfusion during ECMO support poses a significant risk of neurological complications. Careful attention is given to blood flow rates within the ECMO circuit to address this concern. Optimizing blood flow helps ensure sufficient perfusion to vital organs, including the brain. Maintenance of mean arterial pressure (MAP) is another crucial aspect, as adequate blood pressure is essential for preventing hypoperfusion and ischemia. Continuous neurological monitoring is implemented to promptly identify any signs of inadequate perfusion or ischemia, enabling timely interventions to optimize ECMO settings and preserve neurological function [41].

Neurological monitoring: Frequent neurological assessments are integral to ECMO patient management. These assessments provide a comprehensive picture of the patient's neurological status, encompassing mental status, pupillary reactions, and motor responses. Early detection of neurological changes allows for prompt intervention, potentially preventing irreversible damage. Neurological monitoring is particularly crucial during ECMO, where the risk of complications such as emboli or hypoperfusion is heightened. The data collected from these assessments inform clinical decision-making, guide adjustments to ECMO settings, and contribute to a proactive approach to managing neurological complications [30].

Outcomes and survival rates

Success Stories

Long-term survival rates for patients treated with ECMO have been reported in various studies. A study found that the five-year survival rates were 33% for venoarterial (VA)-ECMO and 36% for venovenous (VV)-ECMO. Among patients who survived for 30 days, the five-year survival rates were 73% for VA-ECMO and 71% for VV-ECMO [42]. Another study reported a six-month all-cause mortality rate of 53.0% for patients receiving ECMO for severe COVID-19 [43]. A systematic review and meta-analysis also reported a pooled mortality rate of 48.8% among patients receiving ECMO [44]. However, a recent clinical trial showed significantly higher survival rates among cardiac arrest patients who received ECMO treatment, with patients being six times more likely to survive with ECMO compared to standard treatment [45].

Factors Influencing Patient Outcomes

Several factors influence patient outcomes and survival rates with ECMO. Predictors of increased mortality include advanced age, the time of patient enrollment, the proportion of patients receiving corticosteroids, and the reduced duration of the ECMO run [44]. Additionally, the mode of ECMO (venoarterial vs. venovenous) and the underlying condition being treated (e.g., severe COVID-19, cardiac arrest) can also impact patient outcomes [42,43].

Long-Term Effects

Long-term health-related quality of life (HRQoL) assessments among ECMO survivors have shown persistent concerns, highlighting the importance of longer-term post-discharge follow-up. Despite acceptable five-year survival rates for ECMO patients who survived the initial 30 days, ongoing HRQoL issues were apparent, emphasizing the need for continued monitoring and support for ECMO survivors [42]. While ECMO has shown varying survival rates depending on the underlying condition and patient characteristics, it has demonstrated promising outcomes in specific patient populations, such as those with severe cardiac arrest. Long-term HRQoL assessments have highlighted the need for continued support for ECMO survivors.

Advancements in ECMO technology

Miniaturization and Portable ECMO Devices

Compact ECMO systems: Advancements in miniaturization have revolutionized ECMO technology, developing more compact ECMO systems. These smaller devices offer increased portability, presenting a significant advantage in diverse patient care settings. The portability of compact ECMO systems is particularly beneficial in resource-limited environments where space and infrastructure may be constraints. Additionally, these devices prove invaluable during intra-hospital transport of critically ill patients, providing continuous life support in a more agile and adaptable manner. The development of compact ECMO systems signifies a leap forward in enhancing accessibility and usability, especially in scenarios where traditional, larger ECMO systems might be impractical [20].

Ambulatory ECMO: Portable ECMO devices have enabled the concept of ambulatory ECMO, allowing patients to maintain mobility and engage in rehabilitation activities while receiving life-sustaining support. Ambulatory ECMO represents a transformative approach to patient care during ECMO therapy. By enabling greater mobility, these portable devices enhance the quality of life for patients, fostering a more active and participatory role in their recovery. This contributes to the patient's well-being and aligns with rehabilitation goals, promoting physical activity and functional recovery. Ambulatory ECMO signifies a paradigm shift in ECMO therapy, emphasizing patient-centered care and integrating life support with an active lifestyle [46].

Extracorporeal CO_2_ removal): Innovations in miniaturization have led to the development of dedicated systems focusing on carbon dioxide removal, known as Extracorporeal CO_2_ Removal (ECCO_2_R). Often more compact than traditional ECMO setups, these systems are designed to address primarily hypercapnic respiratory failure. ECCO_2_R devices provide a targeted approach to respiratory support by efficiently removing carbon dioxide from the blood, allowing the patient's lungs to rest and recover. The more compact nature of ECCO_2_R devices enhances their versatility and usability in various clinical scenarios, providing a tailored solution for patients with specific respiratory challenges. This innovation broadens the spectrum of respiratory support options within the ECMO framework, offering a nuanced approach to patient care [47].

Novel Circuit Designs

Closed-loop systems: Closed-loop systems represent a significant advancement in ECMO technology, introducing novel circuit designs that enhance the precision and responsiveness of therapy. These systems continuously monitor critical parameters within the patient's blood, such as oxygen and carbon dioxide levels. Based on this real-time data, the closed-loop system autonomously adjusts the pump speed or oxygenator function to maintain optimal blood gas levels. Automating these adjustments reduces the reliance on manual interventions, streamlining the management of ECMO support. This contributes to fine-tuning therapy, ensuring the patient receives optimal oxygenation and carbon dioxide removal without needing constant manual adjustments. The integration of closed-loop systems represents a leap forward in the efficiency and adaptability of ECMO, offering a more dynamic and responsive approach to patient care [48].

Biocompatible materials: Advances in biomaterials have driven the development of ECMO circuits with enhanced biocompatibility. Traditional ECMO circuits may activate the coagulation cascade and provoke inflammatory responses, leading to complications such as clot formation. Biocompatible materials mitigate these issues by minimizing the body's adverse reactions to the extracorporeal circuit. These materials are designed to be more compatible with the physiological environment, reducing the risk of clotting and inflammation associated with prolonged ECMO support. Using biocompatible materials represents a crucial step in improving the safety and efficacy of ECMO therapy, particularly in cases where prolonged support is necessary [49].

Remote Monitoring and Telemedicine

Real-time monitoring: Real-time monitoring is a pivotal component of advanced ECMO systems, enhancing the ability to remotely assess patient status and circuit function. With the integration of remote monitoring capabilities, healthcare providers can access crucial information in real time from a distance. This includes monitoring vital signs, ECMO circuit parameters, and other relevant clinical data. The real-time nature of this monitoring facilitates prompt identification of any deviations from the desired parameters, allowing for early recognition of issues. Timely intervention based on real-time monitoring improves patient outcomes by promptly addressing complications or adjusting ECMO settings [50].

Teleconsultation: Telemedicine applications in ECMO extend beyond monitoring to include teleconsultation with ECMO specialists. This capability enables healthcare teams in various locations to collaborate seamlessly on patient management strategies. In scenarios where ECMO expertise may not be locally available, teleconsultation provides a valuable avenue for remote collaboration with specialists. This ensures that even in geographically dispersed settings, healthcare providers can access expert guidance, discuss complex cases, and make informed decisions about ECMO management. Integrating teleconsultation enhances the quality and breadth of expertise available to healthcare teams managing ECMO patients [51].

Data analytics and artificial intelligence: Integrating data analytics and artificial intelligence (AI) represents a cutting-edge development in ECMO technology. AI algorithms can analyze vast amounts of patient data generated during ECMO therapy. This analysis goes beyond real-time monitoring and includes identifying patterns, predicting complications, and optimizing ECMO settings for individual patients. By leveraging AI, ECMO systems can move towards personalized patient management, tailoring interventions based on each patient's unique characteristics and responses. The potential for predictive modeling enhances the proactive nature of ECMO care, allowing healthcare providers to anticipate complications and optimize therapeutic strategies more individually [52].

Future directions and research

Emerging Technologies

The future of ECMO is closely linked to developing predictive models and emerging technologies. Experimental studies are shedding new light on the future of ECMO, aiming to address open questions and controversies [53]. Predictive models for ECMO are the subject of ongoing research, with a systematic review highlighting the need for external validation and the uncertainty surrounding the initiation of ECMO [54]. These efforts aim to improve patient selection and outcomes and optimize the use of ECMO in critical care settings.

Clinical Trials and Studies

Research in the field of ECMO is characterized by ongoing clinical trials and studies aimed at addressing challenges and improving patient outcomes. A 10-year bibliometric study and visualization analysis identified research hotspots and trends in ECMO, providing insights into the evolving landscape of ECMO research [55]. Current and future strategies to monitor and manage coagulation in ECMO patients are also a research focus, given the risk of bleeding and thrombosis complications associated with ECMO [56].

Integration With Artificial Intelligence in ECMO Management

While specific information on integrating AI in ECMO management is not provided in the search results, the use of AI in critical care and medical decision-making is a growing area of interest. AI has the potential to enhance ECMO management by analyzing complex patient data, predicting outcomes, and optimizing treatment strategies. However, further research and development are needed to fully understand the potential benefits and challenges of integrating AI into ECMO management. The future of ECMO is closely tied to the development of predictive models, emerging technologies, ongoing clinical trials, and the potential integration of artificial intelligence in ECMO management. These research areas aim to address challenges, improve patient outcomes, and advance the use of ECMO in critical care settings.

## Conclusions

In conclusion, ECMO emerges as a pivotal lifeline in critical care, addressing a diverse range of life-threatening conditions such as respiratory and cardiac failure, trauma, sepsis, and post-cardiotomy shock. Careful patient selection guided by rigorous criteria ensures that ECMO is initiated judiciously, highlighting its multidisciplinary nature. While complications such as bleeding, thrombosis, infection, hemolysis, and neurological issues pose challenges, meticulous monitoring and interventions help navigate these complexities. The landscape of ECMO is continuously evolving through technological advancements, including miniaturization, portable devices, novel circuit designs, and the integration of remote monitoring and telemedicine. This progress enhances the efficacy of ECMO and expands its reach, making it more adaptable and patient-centric. The ongoing impact of ECMO in critical care is underscored by its role in improving survival rates, enabling greater mobility and quality of life through ambulatory support, and facilitating expert consultation through telemedicine. As we reflect on ECMO's journey from historical roots to its current technological sophistication, it becomes clear that its potential for saving lives in the most critical moments is profound and promising, shaping the future landscape of critical care.

## References

[1] Marasco SF, Lukas G, McDonald M, McMillan J, Ihle B (2008). Review of ECMO (extra corporeal membrane oxygenation) support in critically ill adult patients. Heart Lung Circ.

[2] Vyas A, Bishop MA (2023). Extracorporeal membrane oxygenation in adults. https://www.ncbi.nlm.nih.gov/books/NBK576426/.

[3] Rabah H, Rabah A (2022). Extracorporeal membrane oxygenation (ECMO): what we need to know. Cureus.

[4] Bartlett R, Arachichilage DJ, Chitlur M (2024). The history of extracorporeal membrane oxygenation and the development of extracorporeal membrane oxygenation anticoagulation. Semin Thromb Hemost.

[5] Cashen K, Regling K, Saini A (2022). Extracorporeal membrane oxygenation in critically ill children. Pediatr Clin North Am.

[6] Ratnani I, Tuazon D, Zainab A, Uddin F (2018). The role and impact of extracorporeal membrane oxygenation in critical care. Methodist Debakey Cardiovasc J.

[7] Banfi C, Pozzi M, Siegenthaler N (2016). Veno-venous extracorporeal membrane oxygenation: cannulation techniques. J Thorac Dis.

[8] Shelly MP, Nightingale P (1999). ABC of intensive care: respiratory support. BMJ.

[9] Makdisi G, Wang IW (2015). Extra corporeal membrane oxygenation (ECMO) review of a lifesaving technology. J Thorac Dis.

[10] Pavlushkov E, Berman M, Valchanov K (2017). Cannulation techniques for extracorporeal life support. Ann Transl Med.

[11] Bishop MA, Moore A (2023). Extracorporeal Membrane Oxygenation Weaning. https://www.ncbi.nlm.nih.gov/books/NBK570564/.

[12] McRae K, de Perrot M (2018). Principles and indications of extracorporeal life support in general thoracic surgery. J Thorac Dis.

[13] Ambati S, Yandrapalli S (2023). Refractory Hypoxemia and Venovenous ECMO. https://www.ncbi.nlm.nih.gov/books/NBK560516/.

[14] Pappalardo F, Montisci A (2016). Veno-arterial extracorporeal membrane oxygenation (VA ECMO) in postcardiotomy cardiogenic shock: how much pump flow is enough?. J Thorac Dis.

[15] Raman J, Saxena P, Dobrilovic N (2023). ECMO as a bridge to cardiac surgery: stabilizing unstable patients for a definitive procedure. Indian J Thorac Cardiovasc Surg.

[16] Koons B, Siebert J (2020). Extracorporeal membrane oxygenation (ECMO) as a bridge to lung transplantation: considerations for critical care nursing practice. Crit Care Nurse.

[17] Zhang Y, Zhang L, Huang X (2023). ECMO in adult patients with severe trauma: a systematic review and meta-analysis. Eur J Med Res.

[18] Han L, Zhang Y, Zhang Y, Wu W, He P (2019). Risk factors for refractory septic shock treated with VA ECMO. Ann Transl Med.

[19] Lorusso R, Raffa GM, Alenizy K (2019). Structured review of post-cardiotomy extracorporeal membrane oxygenation: part 1-adult patients. J Heart Lung Transplant.

[20] Lequier L, Horton SB, McMullan DM, Bartlett RH (2013). Extracorporeal membrane oxygenation circuitry. Pediatr Crit Care Med.

[21] Mossadegh C (2016). Monitoring the ECMO. Nursing Care and ECMO.

[22] Aokage T, Palmér K, Ichiba S, Takeda S (2015). Extracorporeal membrane oxygenation for acute respiratory distress syndrome. J Intensive Care.

[23] Chiu LC, Kao KC (2021). Mechanical ventilation during extracorporeal membrane oxygenation in acute respiratory distress syndrome: a narrative review. J Clin Med.

[24] Gulack BC, Hirji SA, Hartwig MG (2014). Bridge to lung transplantation and rescue post-transplant: the expanding role of extracorporeal membrane oxygenation. J Thorac Dis.

[25] Joo S, Cho S, Lee JH, Min J, Kwon HW, Kwak JG, Kim WH (2022). Postcardiotomy extracorporeal membrane oxygenation support in patients with congenital heart disease. J Chest Surg.

[26] Rubin GD, Ryerson CJ, Haramati LB (2020). The role of chest imaging in patient management during the covid-19 pandemic: a multinational consensus statement from the Fleischner Society. Radiology.

[27] Hussey PT, von Mering G, Nanda NC, Ahmed MI, Addis DR (2022). Echocardiography for extracorporeal membrane oxygenation. Echocardiography.

[28] Bercovitz RS (2018). An introduction to point-of-care testing in extracorporeal circulation and LVADs. Hematology Am Soc Hematol Educ Program.

[29] Doo I, Staub LP, Mattke A, Haisz E, Seidler AL, Alphonso N, Schlapbach LJ (2021). Diagnostic accuracy of infection markers to diagnose infections in neonates and children receiving extracorporeal membrane oxygenation. Front Pediatr.

[30] Aboul-Nour H, Jumah A, Abdulla H, Sharma A, Howell B, Jayaprakash N, Gardner-Gray J (2023). Neurological monitoring in ECMO patients: current state of practice, challenges and lessons. Acta Neurol Belg.

[31] Jayaraman AL, Cormican D, Shah P, Ramakrishna H (2017). Cannulation strategies in adult veno-arterial and veno-venous extracorporeal membrane oxygenation: techniques, limitations, and special considerations. Ann Card Anaesth.

[32] Kumar G, Maskey A (2021). Anticoagulation in ECMO patients: an overview. Indian J Thorac Cardiovasc Surg.

[33] Burstein B, Wieruszewski PM, Zhao YJ, Smischney N (2019). Anticoagulation with direct thrombin inhibitors during extracorporeal membrane oxygenation. World J Crit Care Med.

[34] Kim HS, Park S (2017). Blood transfusion strategies in patients undergoing extracorporeal membrane oxygenation. Korean J Crit Care Med.

[35] Abruzzo A, Gorantla V, Thomas SE (2022). Venous thromboembolic events in the setting of extracorporeal membrane oxygenation support in adults: a systematic review. Thromb Res.

[36] Staessens S, Moussa MD, Pierache A (2022). Thrombus formation during ECMO: insights from a detailed histological analysis of thrombus composition. J Thromb Haemost.

[37] O'Grady NP, Alexander M, Burns LA (2011). Guidelines for the prevention of intravascular catheter-related infections. Clin Infect Dis.

[38] Peña-López Y, Machado MC, Rello J (2023). Infection in ECMO patients: changes in epidemiology, diagnosis and prevention. Anaesth Crit Care Pain Med.

[39] Appelt H, Philipp A, Mueller T (2020). Factors associated with hemolysis during extracorporeal membrane oxygenation (ECMO)-Comparison of VA- versus VV ECMO. PLoS One.

[40] Kazmi SO, Sivakumar S, Karakitsos D, Alharthy A, Lazaridis C (2018). Cerebral pathophysiology in extracorporeal membrane oxygenation: pitfalls in daily clinical management. Crit Care Res Pract.

[41] Khanduja S, Kim J, Kang JK (2023). Hypoxic-ischemic brain injury in ECMO: Pathophysiology, neuromonitoring, and therapeutic opportunities. Cells.

[42] Rossong H, Debreuil S, Yan W, Hiebert BM, Singal RK, Arora RC, Yamashita MH (2023). Long-term survival and quality of life after extracorporeal membrane oxygenation. J Thorac Cardiovasc Surg.

[43] Biancari F, Mariscalco G, Dalén M (2021). Six-month survival after extracorporeal membrane oxygenation for severe COVID-19. J Cardiothorac Vasc Anesth.

[44] Ling RR, Ramanathan K, Sim JJ (2022). Evolving outcomes of extracorporeal membrane oxygenation during the first 2 years of the COVID-19 pandemic: a systematic review and meta-analysis. Crit Care.

[45] (2023). mende434: World’s first-ever ECMO-based clinical trial shows six times higher survival rates among cardiac arrest patients. https://med.umn.edu/news/worlds-first-ever-ecmo-based-clinical-trial-shows-six-times-higher-survival-rates-among-cardiac-arrest-patients.

[46] Lindholm JA (2018). Ambulatory veno-venous extracorporeal membrane oxygenation. J Thorac Dis.

[47] Titus A, Sanghavi DK (2023). Extracorporeal Carbon Dioxide Removal. https://www.ncbi.nlm.nih.gov/books/NBK556004/.

[48] Conway RG, Berk ZB, Zhang J, Li T, Tran D, Wu ZJ, Griffith BP (2020). Evaluation of an autoregulatory ECMO system for total respiratory support in an acute ovine model. Artif Organs.

[49] Roberts TR, Garren MR, Handa H, Batchinsky AI (2020). Toward an artificial endothelium: development of blood-compatible surfaces for extracorporeal life support. J Trauma Acute Care Surg.

[50] Aguirre AD, Shelton KT (2022). Remote monitoring in the use of extracorporeal membrane oxygenation and acute mechanical circulatory support. Curr Opin Crit Care.

[51] Kemp Van Ee S, McKelvey H, Williams T (2022). Telemedicine intensive care unit (Tele-ICU) implementation during COVID-19: a scoping review. Cureus.

[52] Khan ZF, Alotaibi SR (2020). Applications of artificial intelligence and big data analytics in m-health: a healthcare system perspective. J Healthc Eng.

[53] Giani M, Arcadipane A, Martucci G (2021). Challenges in the extracorporeal membrane oxygenation era. Membranes (Basel).

[54] Giordano L, Francavilla A, Bottio T (2023). Predictive models in extracorporeal membrane oxygenation (ECMO): a systematic review. Syst Rev.

[55] Wang H, Deng S, Fan X, Li J, Tang L, Li Y, Yu B (2021). Research trends and hotspots of extracorporeal membrane oxygenation: a 10-year bibliometric study and visualization analysis. Front Med (Lausanne).

[56] Zeibi Shirejini S, Carberry J, McQuilten ZK, Burrell AJ, Gregory SD, Hagemeyer CE (2023). Current and future strategies to monitor and manage coagulation in ECMO patients. Thromb J.

